# Stellenwert der Biopsie in der Diagnostik chronischer Wunden – ein Positionspapier der Initiative Chronische Wunden (ICW) e. V.

**DOI:** 10.1007/s00105-023-05259-7

**Published:** 2023-12-01

**Authors:** C. Erfurt-Berge, A. Bültemann, V. Gerber, M. Motzkus, J.-D. Rembe, J. Dissemond

**Affiliations:** 1 Hautklinik, Uniklinikum Erlangen, Ulmenweg 18, 91054 Erlangen, Deutschland; 2 Klinik für Gefäß- und Viszeralchirurgie, Asklepios Klinikum Harburg, Hamburg, Deutschland; 3 Initiative Chronische Wunden (ICW) e. V., Quedlinburg, Deutschland; 4 ZentralesWundmanagement, Evangelisches Krankenhaus Mülheim, Mülheim/Ruhr, Deutschland; 5 Klinik für Gefäß- und Endovaskularchirurgie, Universitätsklinikum Düsseldorf, Düsseldorf, Deutschland; 6 Klinik und Poliklinik für Dermatologie, Venerologie und Allergologie, Universitätsklinikum Essen, Essen, Deutschland

**Keywords:** Ulcus cruris, Differenzialdiagnose, Probebiopsie, Histologie, Mikrobiologie, Leg ulcer, Differential diagnosis, Tissue sample, Histology, Microbiology

## Abstract

Die Abklärung chronischer Wunden ist essenziell für die Einleitung einer kausaltherapeutischen Behandlung. Zur diagnostischen Einordnung der Wundgenese kann es erforderlich sein, eine Biopsie zur histologischen und/oder mikrobiologischen Aufarbeitung zu entnehmen. Besteht klinisch Verdacht auf eine spezifische Ursache der Wunde wie eine Neoplasie, eine entzündliche Dermatose oder eine erregerbedingte Wunde, so ist unverzüglich eine Biopsie zur weiteren Diagnostik erforderlich. Zeigt sich unter einer adäquat erscheinenden Kausaltherapie kein zufriedenstellendes Therapieansprechen der Ulzeration, so ist spätestens nach 12 Wochen eine Biopsie zur weiteren Evaluation empfohlen. Die Wahl der richtigen Entnahmetechnik, die weitere Lagerung, der Transport und die Aufarbeitung sind dabei ebenso entscheidend für ein verwertbares Ergebnis wie die möglichst spezifische Fragestellung an das diagnostische Labor.

## Abstract (en)

Diagnostic assessment of chronic wounds is essential for the initiation of causal therapeutic treatment. For diagnostic classification of the wound genesis, it may be necessary to take a tissue sample for histological and/or microbiological processing. If there is clinical suspicion of a specific cause of the wound such as a neoplasm, an inflammatory dermatosis or a pathogen-induced wound, a tissue sample for further diagnosis is required immediately. If the ulceration does not respond sufficiently to adequate causal therapy, a tissue sample for further evaluation is recommended after 12 weeks. The choice of the correct sampling technique, further storage, transport and processing are just as decisive for a reliable result as the specific question for the diagnostic laboratory.

Die histologische Untersuchung einer Biopsie stellt einen wichtigen Eckpfeiler in der Differenzialdiagnostik chronischer Wunden dar. Insbesondere seltene Ursachen chronischer Wunden können in vielen Fällen gerade erst durch Berücksichtigung des histopathologischen Befundes festgestellt werden. Auch die mikrobiologische Diagnostik ist in bestimmten Fällen eher anhand von Biopsien im Gegensatz zu den ansonsten oft ausreichenden Wundabstrichen durchzuführen [22]. Dem Dermatologen/der Dermatologin kommt dabei insbesondere in der Diagnostik seltener Ursachen chronischer Wunden eine Schlüsselfunktion zu.

## Indikationsstellung für eine Biopsie

Die Entnahme einer Biopsie bei Menschen mit einer chronischen Wunde ist eine wichtige und häufig unverzichtbare Maßnahme, um die Wunde nosologisch einordnen zu können. Einerseits kann bereits initial aufgrund der Konfiguration, Wundumgebung und Lokalisation der Wunde oder aufgrund einer typischen Anamnese eine bestimmte Differenzialdiagnose im Raum stehen, welche zur weiteren Diagnostik eine Aufarbeitung einer Biopsie notwendig macht. Aber auch eine Wunde, die trotz Umsetzung adäquat erscheinender Diagnostik- und Therapiemaßnahmen keine Abheilungstendenz zeigt, beispielsweise unter dem initialen Verdacht auf eine vaskulär bedingte Wunde, kann dazu führen, dass die Entscheidung für eine Biopsie als weiterer diagnostischer Schritt fällt (Tab. 1).

**Table 1:** 

Diagnostische Hinweise für die Biopsie	Vermutete Diagnose
Atypische^a^ Anamnese	Neoplasie, erregerbedingte Wunde
Atypische^a^ Lokalisation	Neoplasie, erregerbedingte Wunde, spezifische Dermatose, z. B. Pyoderma gangraenosum, ulzerierte Necrobiosis lipoidica
Atypische^a^ Konfiguration, z. B. unregelmäßiger Wundrand, erhabener Wundrand, derber Randwall, Hypergranulation, bizarre Ausläufer, ungewöhnliche Tiefe	Neoplasie, Vaskulopathie, Kalziphylaxie, Ulcus cruris hypertonicum Martorell, spezifische Dermatose Aktinomykose (Lokalisation v. a. im Gesicht, ggf. auch Fistelinhalt untersuchen) Mykobakteriose (Lokalisation v. a. Hände/Unterarme)
Therapieresistenz (trotz scheinbar adäquater Therapie)	Neoplasie
Purpura in der Wundumgebung	Vaskulitis
Livider Randsaum	Pyoderma gangraenosum, Vaskulitis, Vaskulopathie
Ungewöhnlich starke Schmerzen, ungewöhnlicher Geruch, Blutungstendenz	Neoplasie, erregerbedingte Wunde, sekundäre Weichgewebeinfektion

^a^Per se gibt es für bestimmte Differenzialdiagnosen oft relativ typische Kriterien, welche jedoch beim Großteil der vornehmlich vaskulär bedingten Ulcera crurum nicht zutreffen und daher hier als „atypisch“ bezeichnet sind

Die entnommene Biopsie kann durch unterschiedliche Methoden untersucht werden. Dies hängt von der vermuteten (Differenzial‑)Diagnose ab. Entscheidend ist, dass bereits in der Anforderung der entsprechenden Untersuchungen gezielt eine Verdachtsdiagnose geäußert werden sollte, um eine korrekte Diagnostik zu ermöglichen.

Während manche Diagnosen durch Inspektion und Anamnese bereits direkt vermutet und mithilfe einer histologischen Gewebeaufarbeitung bestätigt werden können, ist die Entscheidung für eine Biopsie bei fehlendem Therapieansprechen einer anhaltenden Diskussion über den Zeitrahmen ausgesetzt, nach dessen Verstreichen eine Biopsie in jedem Fall erforderlich sei. Das gilt insbesondere im Hinblick auf den Ausschluss einer malignen Wunde. Bei Auftreten der in der Tab. 1 genannten klinischen Veränderungen der Wundkonfiguration sollte umgehend eine Biopsie oder besser mehrere Biopsien erfolgen [25]. Fehlen diese klinischen Verdachtsmomente, so ist die Frage schwieriger zu beantworten, wann eine Biopsie im Falle einer ausbleibenden Besserung trotz adäquat erscheinender Therapiemaßnahmen zu empfehlen ist. So empfehlen beispielsweise Miller et al. [16], venöse Ulzera älter als 12 Monate, bei denen eine adäquate Therapie inklusive Kompressionstherapie bereits umgesetzt wurde, bei Erstvorstellung im spezialisierten Wundzentrum umgehend bioptisch abzuklären. Sollten die Therapiemaßnahmen noch nicht adäquat umgesetzt worden sein, so sollte dies laut den Autoren über 3 Monate erfolgen und bei dann weiterhin ausbleibendem Therapieerfolg eine Biopsie durchgeführt werden. Obwohl sich in der Literatur unterschiedliche Angaben zur Zeitspanne bis zur Biopsie finden [17], empfehlen die meisten Autoren [23] eine Biopsie bei anhaltender Therapieresistenz spätestens 3 Monate nach konsequenter Therapie der Verdachtsdiagnose. Allerdings basieren diese Empfehlungen nicht auf einer wissenschaftlich geprüften Evidenz, sondern entsprechen einer Expertenempfehlung, da randomisiert kontrollierte Studien mit ausreichend großer Patientenzahl zur Thematik gerade bei seltenen Ursachen chronischer Wunden fehlen. Zudem sollte beachtet werden, dass für bestimmte chronische Wunden keine kurative Behandlungsoption vorliegt und hier somit auch keine Befundbesserung trotz adäquater Therapie zu erwarten ist [6].

Nachfolgend werden wichtige Differenzialdiagnosen und deren Besonderheiten im Hinblick auf die histopathologische Untersuchung differenziert besprochen. Die Tab. 2 zeigt beispielhaft wichtige Differenzialdiagnosen als Übersicht.

**Table 2:** 

Tumorerkrankungen
Kutane Tumore
Kutane Metastasen
Organtumor, z. B. Mammakarzinom
Entzündliche Erkrankungen
Pyoderma gangraenosum (histologischer Befund nur hinweisgebend)
Ulzerierte Necrobiosis lipoidica
Bullöse Autoimmundermatosen
Kutane Vaskulitiden
Vaskulopathien
Livedovaskulopathie
Kalziphylaxie
Ulcus cruris hypertonicum Martorell
Infektiöse Erkrankungen (hier Erregernachweis mittels Gewebe-PCR empfohlen)
Leishmaniose
Atypische Mykobakteriose (*M. marinum, M. chelonae*, Buruli-Ulcus [*M. ulcerans*])
Mykobakteriose (Hauttuberkulose)
Lepra
Sporotrichose
Myzetom (bakteriell bedingt, Pilzinfektion)
Lues maligna (zusätzlich andere Nachweistechniken für Erreger möglich und bei einschlägiger Anamnese primär durchzuführen)

### Maligne Wunden

Die Diagnostik kutaner Neoplasien unter dem klinischen Bild einer chronischen Wunde ist eine Domäne der (Dermato‑)Histopathologie. Gerade für maligne Transformationen primär nichtmaligner Wunden (Marjolin-Ulkus) spielt die Biopsie zur frühzeitigen Erkennung eine entscheidende Rolle. Klinische Zeichen wie ein derber Randwall, knotige Veränderungen am Wundgrund, Wundrand oder in der weiteren Wundumgebung, eine für die angenommene Arbeitsdiagnose ungewöhnliche Lokalisation oder auch starke Schmerzen können dabei erste klinisch hinweisende Faktoren für eine Neoplasie sein [10]. Zu unterscheiden sind dabei primär kutane Neoplasien wie Basalzellkarzinome, Plattenepithelkarzinome oder auch Melanome von kutanen Ulzerationen durch solide Organneoplasien wie etwa beim Mammakarzinom [24]. Wenn Infiltrate bestimmter Zellreihen histologisch auffallen, kann dies Hinweise auf systemische hämatoonkologische Erkrankungen wie Leukämien oder Lymphome geben [21], welche primär durch eine kutane Beteiligung symptomatisch geworden sind. Auch ulzerierende Hautmetastasen solider Tumoren, z. B. des Prostatakarzinoms, müssen differenzialdiagnostisch bedacht werden [14]. Zwar ist die Prävalenz gerade als Differenzialdiagnose zum vaskulären Ulcus cruris niedrig [9], jedoch sollte bei dringendem klinischem Verdacht und initial unauffälligem Biopsiebefund eine Re-Biopsie bei fehlendem Therapieansprechen in Erwägung gezogen werden [3]. Gerade für die Erfassung einer Ausbreitung einer Neoplasie sind meist mehrere Biopsien aus unterschiedlichen Arealen der Wunde empfehlenswert, z. B. Zentrum der Wunde und Wundrand. Neben der histopathologischen Standardaufarbeitung, z. B. mit HE(Hämatoxylin-Eosin)-Färbung, sind gezielte Färbungen der für die jeweilige Neoplasieart typischen Marker im Rahmen einer immunhistochemischen Anfärbung indiziert. Die weitere Patientenversorgung nach gestellter Diagnose der Neoplasie richtet sich je nach der Entität. Dabei sind zunächst meist entsprechende Staginguntersuchungen zu initiieren, und das weitere Procedere ist im Idealfall in einem interdisziplinären Tumorboard zu besprechen.

Zu beachten ist, dass seltenere Infektionen der Haut die klinischen Zeichen einer Neoplasie wie einen derben Randwall aufweisen können und daher differenzialdiagnostisch ebenfalls abgegrenzt werden müssen.

### Entzündliche Dermatosen

**Figure 1:**
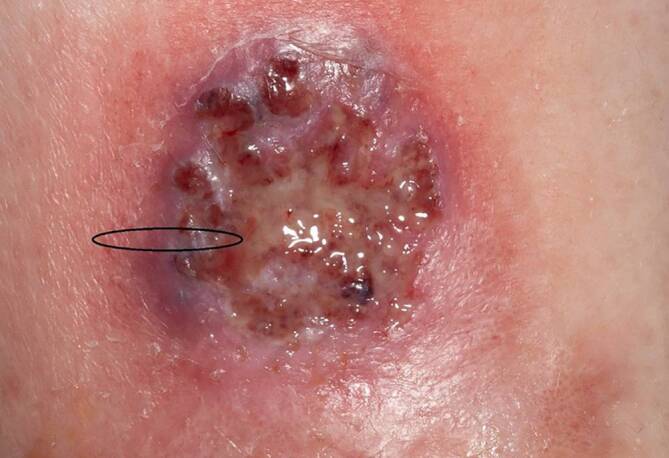

Entzündliche Dermatosen können sich in vielen Fällen mit Ulzerationen als Symptom äußern. Es sollte eine ausführliche Anamnese über Lokalisation und Dynamik der ersten aufgetretenen Hautveränderungen durchgeführt werden. Neben einem meist typischen klinischen Bild kann in vielen Fällen auch mit entsprechender Labordiagnostik, z. B. Nachweis spezifischer Antikörper, die Diagnose untermauert werden. Nicht immer jedoch existieren spezifische Marker, wie beim Pyoderma gangraenosum (PG). Hier ist der Nachweis einer neutrophilen Vaskulitis in der histologischen Untersuchung einer von mehreren Wegweisern zur richtigen Diagnose. Im PARACELSUS Score, einem validierten diagnostischen Tool bei Verdacht auf PG, ist der histologische Befund einer suppurativen Inflammation insgesamt nur mit 1 Punkt bewertet. Die Diagnose PG gilt als wahrscheinlich ab einem Punktwert von 10 Punkten [13]. Die Biopsie sollte hierbei vom entzündlich aktiven Randsaum der Wunde genommen werden. Besonders hinzuweisen ist der Patient auf das Auftreten eines möglichen Pathergiephänomens im Rahmen der Biopsie mit konsekutiver Vergrößerung einer vorbestehenden Wunde. Da die Diagnose eines PG jedoch meist bereits klinisch gestellt wird und unmittelbar nach der Gewebeentnahme eine immunsuppressive Therapie [1] eingeleitet wird, wird das Pathergiephänomen infolge einer Biopsie klinisch nur selten beobachtet. Auch bei Ulzerationen, welche im Rahmen von kutanen Vaskulitiden auftreten, ist der Zielbereich für eine Gewebeentnahme der Wundrand als Ort der höchsten entzündlichen Aktivität bzw. eine möglichst frisch aufgetretene Primäreffloreszenz, fallweise auch fernab der aktuellen Ulzeration. Da Vaskulitiden nach der aktuellen Klassifikation auch nach der Größe der beteiligten Gefäße eingeteilt werden [7], darf die Biopsie auch nicht zu klein bzw. zu oberflächlich entnommen werden, um hier auch entsprechend Gefäße mit zu erfassen. Es empfiehlt sich eine spindelförmige Biopsie mindestens bis in die tiefe Dermis (Abb. 1).

Andere inflammatorische Dermatosen, die zu Ulzerationen führen können, sind z. B. bullöse Autoimmundermatosen, Necrobiosis lipoidica, Pannikulitiden oder Kollagenosen. Die Lokalisation der Gewebeentnahme orientiert sich dabei am Vorliegen von Primäreffloreszenzen, z. B. intakter Blasen, kann also auch fernab einer Ulzeration liegen. Für bestimmte Erkrankungen oder Untersuchungstechniken wie die direkte Immunfluoreszenz (DIF) kann es auch sinnvoll sein, eine Biopsie periläsional, also aus der klinisch intakten Haut neben einer Hautveränderung zu entnehmen, da hier die noch initialen Entzündungsprozesse auf mikroskopischer Ebene nachweisbar sind.

Zusätzlich zur klassischen histopathologischen Aufarbeitung sind v. a. bei entzündlichen Erkrankungen als Differenzialdiagnose chronischer Wunden weitere Untersuchungen möglich. Hierzu zählt insbesondere die DIF, bei der in vivo gebundene Autoantikörper direkt im Gewebe mithilfe fluoreszierender Antikörper sichtbar gemacht werden können. Dies setzt die Kenntnis der Zielstruktur für die jeweilige Erkrankung voraus, wie sie etwa bei bullösen Dermatosen vorliegt. Die Gewebeuntersuchung ergänzt sich dabei mit Anamnese, klinischem Bild und fallweise spezifischen Labormarkern zum Gesamtbild der Diagnostik der Dermatose.

### Vaskulopathien

Fallweise ist es schwierig, sekundär inflammatorische Vaskulitiden infolge einer primären Vaskulopathie oder umgekehrt Obliterationen infolge einer Vaskulitis (z. B. bei septischer Vaskulitis) im zeitlichen Verlauf beider Erkrankungen voneinander zu unterscheiden. Hier sind der Zeitpunkt der Biopsie und die Dauer des Bestehens der zu biopsierenden Veränderung von entscheidender Bedeutung. Obliterative Vaskulopathien sind gekennzeichnet durch Proliferation der Gefäßwand z. B. im Rahmen maligner Prozesse, Embolisation von Gefäßen durch Ablagerungen oder Koagulopathien mit Obliteration infolge von Thromben [18].

Weitere Krankheitsbilder in dieser Kategorie sind die Kalziphylaxie, bei der die typische Trias aus stark schmerzhaften Ulzerationen, Livedozeichnung in der Wundumgebung und histologischer Nachweis der arteriolären Mediaverkalkung in der van Kossa-Färbung zur Diagnose führt [8]. Obwohl auch hier eine Vergrößerung der Wunden durch die Biopsie von einigen Autoren gefürchtet wird, ist die Biopsie zur Abgrenzung anderer Vaskulopathien empfohlen.

Ein weiterer klassischer Vertreter der mit chronischen Wunden einhergehenden Vaskulopathien ist die Livedovaskulopathie. Im Jahr 2021 erschien hierzu erstmalig eine S1-Leitlinie [20], die die Entnahme einer Spindelbiopsie aus dem Randbereich des betroffenen Areals empfiehlt.

Eine Immunfluoreszenzdiagnostik ist bei Vaskulopathien nicht zielführend, kann jedoch erforderlich sein, um entzündliche Erkrankungen wie Vaskulitiden abzugrenzen.

## Neue Verfahren der nichtinvasiven Bildgebung

In den letzten Jahren haben neue bildgebende Verfahren in die dermatologische Diagnostik Einzug gehalten. Hierzu zählt unter anderem die optische Kohärenztomographie, eine nichtinvasive bildgebende Technik, die In-vivo-Bilder der obersten Hautschichten bis in die mittlere Dermis liefern kann [26]. Eine andere Technik ist die konfokale Lasermikroskopie. Fallweise können diese neuen Techniken die histologische Aufarbeitung einer Biopsie ergänzen. Erste Versuche laufen auch zur Diagnostik chronischer Ulzera [12]. Da hier die Ursache aber vielfach in einer tieferen dermalen Schicht oder in der Subkutis gelegen ist, reichen die bisherigen Verfahren noch nicht flächendeckend für alle Differenzialdiagnosen chronischer Wunden aus.

## Gewebediagnostik bei infektiös bedingten Wunden

Neben der histopathologischen Untersuchung kann eine mikrobiologische Diagnostik an Gewebematerial erforderlich sein, um eine infektiöse Ursache einer chronischen Wunde oder tiefer im Gewebe liegende Erreger zu diagnostizieren. In der täglichen Praxis wird eine bakterielle Kolonisation oder ein Erreger einer lokalen Infektion in der Regel durch einen Wundabstrich nachgewiesen [4]. In einer Studie an chronischen Wunden unterschiedlicher Genese wurde gezeigt, dass eine lokalisierte Abstrichentnahme mit Levine-Technik einer Erregerkultur aus Biopsiematerial vom oberflächlichen Wundgrund an gleicher Stelle nicht unterlegen war [11]. Da durch eine Biopsie jedoch das vollständige Kolonisationsspektrum einer chronischen Wunde nicht dargestellt werden kann, ist die Indikation zum Einsatz der Biopsie zur Bestimmung von Erregern in chronischen Wunden streng zu stellen. Es bleibt vorab zu klären, welches Ziel mit einem Erregernachweis in der Biopsie erreicht werden soll. Zur Erfassung der bakteriellen Kolonisation einer Wunde bleibt der Abstrich der Biopsie überlegen. Bei einer vermuteten Weichteilinfektion beim diabetischen Fußulkus jedoch empfehlen die Leitlinien eine Erregerkultur aus Gewebe (Biopsat oder Kürettagematerial) [15]. Im Einzelfall kann es bei septischen Komplikationen entscheidend sein, dass vor Beginn der Antibiotikatherapie eine korrekte Diagnostik der relevanten Erreger erfolgt war.

Zusätzlich gibt es bestimmte Erreger, die allein durch Anzucht oder Nachweis mittels PCR (Polymerase-Ketten-Reaktion) aus Gewebe nachzuweisen sind. Eine Untersuchung aus einem Wundabstrich mithilfe eines Abstrichträgers [22] ist dafür nicht ausreichend. Hierzu zählen neben bestimmten Bakterien auch Pilze oder Parasiten als ursächliche Erreger einer chronischen Wunde. Seltenere Infektionserreger wie Hauttuberkulose oder Aktinomykose [19] können dabei auch klinisch das Bild eines ulzerierten malignen Tumors imitieren. Sie treten eher an ungewöhnlichen Lokalisationen wie Händen, Armen oder im Gesicht auf. Fallweise ist eine weitere bildgebende Untersuchung erforderlich, um extrakutane Infektionsherde zu detektieren. Für die Gewebeprobe empfiehlt sich zur korrekten Anforderung und Auswahl des geeigneten Transportmediums in solchen Fällen eine vorherige Rücksprache mit dem mikrobiologischen Labor. Entscheidend ist, dass die wegweisende Untersuchung nur bei gezielter Fragestellung erfolgen kann. Dies kann auch dann gegeben sein, wenn mehrfache Abstriche keine klaren oder plausiblen Ergebnisse erbringen und auch der histologische Befund fraglich ist, wie dies z. B. bei *Mycobacterium-marinum*-Infektionen der Fall sein kann.

## Empfehlungen für die Praxis

- *Bei Auffälligkeiten in der Anamnese, der Lokalisation oder der Konfiguration der Wunde sollte unverzüglich eine Biopsie zum Ausschluss eines malignen Geschehens erfolgen*.
- *Bei Verdacht auf eine spezifische Ursache einer chronischen Wunde, welche durch eine histopathologische Untersuchung diagnostisch eingeordnet werden kann, sollte unverzüglich eine Biopsie erfolgen. Hierzu zählen insbesondere entzündliche Dermatosen als Ursache chronischer Wunden. Die Einsendung von Gewebe für weiterführende Untersuchungen (z.* *B. Immunfluoreszenz) sollte unter Beachtung der Verdachtsdiagnose zielgerichtet erfolgen*.
- *Bei Verdacht auf eine erregerbedingte Wunde, deren Erreger idealerweise im Gewebe nachgewiesen werden kann, sollte unter Berücksichtigung der Anamnese und der klinischen Befunde bei entsprechendem Verdacht unverzüglich eine Biopsie zur mikrobiologischen Untersuchung unter genauer Mitteilung der infrage kommenden Erreger in das entsprechende Labor übersandt werden*.
  *Der Begriff „unverzüglich“ kann in seinem juristischen Verständnis dabei auch eine Zeit von mehreren Tagen umfassen, falls beispielsweise eine gesonderte Terminvereinbarung für eine Biopsie erforderlich ist*.
- *Bei einer ausbleibenden Befundbesserung trotz adäquat erscheinender Umsetzung aller kausal- und lokaltherapeutischen Maßnahmen in Bezug auf die jeweilige Arbeitsdiagnose sollte spätestens nach 12 Wochen eine Biopsie zur weiterführenden Diagnostik erfolgen*.

## Planung und Durchführung einer Biopsie

Wenn die Indikation für die Entnahme einer Biopsie gestellt wurde, so sind vor der Durchführung weitere Überlegungen zu treffen (Tab. 3). Es müssen ein geeigneter Eingriffsraum und das notwendige Instrumentarium zur Verfügung stehen. Ähnlich wie bei der Planung eines chirurgischen Débridements [5] müssen Überlegungen zu Analgesie, Anästhesie, technischer Durchführung und postoperativer Versorgung getroffen werden. Neben der genauen Lokalisation der Probeentnahme in Bezug auf die Wunde (Wundgrund, Wundrand, Wundumgebung) in Abhängigkeit der vermuteten Differenzialdiagnose und Fragestellung an das histopathologische Labor müssen je nach Größe und Tiefe der geplanten Biopsie das Instrumentarium (Stanzbiopsie, Skalpell) und Nahtmaterial ausgesucht werden. In den meisten Fällen bedarf es einer Spindelbiopsie unter Mitnahme der Subkutis und bei besonderen Fragestellungen auch der Faszie zur eindeutigen histopathologischen Beurteilung. Nicht immer ist zwingend eine Naht am Wundrand der Entnahmestelle notwendig, teilweise aufgrund ausgeprägter Dermatosklerose oder Atrophie auch nicht möglich. Hier kann die Entnahmestelle wie die Gesamtwunde mit zeitgemäßen Verbandmitteln versorgt und eine sekundäre Wundheilung angestrebt werden. Bei leichten Blutungen kann beispielsweise ein Calciumalginat oder Kollagen eingesetzt werden. Je nach geplanter Untersuchung muss das geeignete Transportmedium gewählt werden. Für mikrobiologische Fragestellungen empfiehlt sich ein Transport in 0,9 % NaCl-Lösung oder in einer mit physiologischer Kochsalzlösung angefeuchteten sterilen Kompresse im entsprechenden Diagnostikröhrchen. Für die klassische histologische Aufarbeitung erfolgt zumeist ein Transport im Formalin-Medium. Spezifische Fragestellungen, wie eine Untersuchung mittels Immunfluoreszenz bedürfen anderer Medien, wie z. B. Michel’s Medium. Die Indikation zu den unterschiedlichen Untersuchungen wird in diesem Artikel bei den jeweiligen Diagnosen gesondert besprochen. Eine Rücksprache mit dem entsprechenden Labor ist bei Unsicherheiten bereits im Vorfeld der Gewebeentnahme angeraten. Ebenso ist es hilfreich, dem befundenden histopathologischen oder mikrobiologischen Labor vorab weiterführende Informationen über den Fall und ggf. eine Fotodokumentation der Wunde zukommen zu lassen. So können gemeinsam die geeignete Entnahmetechnik und Lokalisation festgelegt werden. Lokale oder systemische Vortherapien, z. B. eine immunsuppressive Medikation oder ein Antibiotikum, können von Relevanz für das Befundergebnis sein. Auch Vorgaben hinsichtlich der Temperatur bei Lagerung oder Transport sind je nach geplanter Untersuchung zu beachten. Das gilt insbesondere dann, wenn eine Biopsie nicht unmittelbar nach Entnahme ins Labor transportiert werden kann.

**Table 3:** 

Parameter zur Planung einer Biopsie	
Technik der Entnahme	Stanzbiopsie, Inzisionsbiopsie mit Skalpell („Spindelbiopsie“)
Materialien	Instrumentarium, Lokalanästhesie, sterile Tücher und Handschuhe, Nahtmaterial, Verbandmaterial, Transportgefäße inklusive passendes Medium, Anforderungsschein, blutstillende Wundauflagen, z. B. Calciumalginat, Cellulose
Transportmedium	Je nach geplanter Untersuchung
Lagerungsmöglichkeiten, z. B. Temperaturvorgaben	Je nach geplanter Untersuchung
Anforderung an das Labor	Spezielle Aufarbeitung/Schnitt je nach Fragestellung; Erregernachweise; Einsatz spezieller Färbetechniken je nach Angabe einer Verdachtsdiagnose, z. B. van Kossa-Färbung bei Verdacht auf Kalziphylaxie oder Ziehl-Neelsen-Färbung bei Mykobakterien
Tiefe der Biopsie	In Abhängigkeit der zu beurteilenden Strukturen, z. B. dermale Gefäße, Pannikulus/Subkutis
Größe der Biopsie	In Abhängigkeit der erforderlichen, unterschiedlichen Untersuchungstechniken, z. B. Histopathologie, Immunfluoreszenz, Mikrobiologie
Anzahl der zu entnehmenden Biopsien	Aus unterschiedlichen Arealen, z. B. Wundrand und Zentrum der Wunde je nach Fragestellung
Anästhesie	Oberflächenanästhesie, lokale Infiltrationsanästhesie, perioperative Analgesie
Lokalisation	Wundumgebung, z. B. bei Livedo racemosa; Primäreffloreszenz, z. B. bei Vaskulitis; Wundrand, zonale Biopsie mit Längsaufarbeitung in der Histologie
Patienteneigene Faktoren	Aufklärungsbedarf, Schmerzerleben, Angst, postoperative Überwachung

## Risiken einer Gewebeentnahme

Vor der Durchführung einer Biopsie muss eine ärztliche Aufklärung über den Eingriff mit entsprechender Bedenkzeit für den Patienten durchgeführt werden. Dabei werden die allgemeinen Risiken einer Biopsie wie Blutung, Infektionsgefahr, Nervenverletzung, aber auch Auftreten von Wundheilungsstörungen angesprochen. Gerade bei chronischen Wunden besteht die Gefahr, dass durch die Entnahme von Gewebe eine neue Wunde oder eine Vergrößerung der vorbestehenden Wunde auftreten kann. Bei entzündlichen Erkrankungen wie dem PG kann dieses Risiko im Sinne eines Pathergiephänomens erhöht sein, insbesondere, wenn nicht unmittelbar nach Biopsie eine initiale immunsuppressive Therapie eingeleitet wird. Häufig ist es aufgrund einer starken Spannung im Bereich der Wundränder nicht möglich, diese mit einer Naht wieder vollständig zu adaptieren. In diesen Fällen ist eine sekundäre Wundheilung anzustreben, und es kann zu einer verzögerten Wundheilung kommen. Der Patient ist zudem nach bekannten Allergien zu befragen, um auf die Gefahr einer allergischen Reaktion z. B. auf Lokalanästhetika aufmerksam zu machen. Postoperativ sollte der Patient eine Extremität, an der die Biopsie durchgeführt wurde, hochlagern und entlasten. Fallweise kann eine zeitweise postoperative Überwachung des Patienten, z. B. bei Antikoagulation erforderlich sein. In der Regel ist es für die Entnahme einer Biopsie aufgrund des üblicherweise geringen Blutungsrisikos nicht erforderlich, eine Antikoagulation zu pausieren. Das Anlegen eines Kompressionsverbandes kann – unter Beachtung von Kontraindikationen – postoperativ ebenfalls hilfreich zur Vermeidung von Blutungen und/oder Hämatomen sein. Sollten Blutungskomplikationen auftreten, können Hämostyptika wie eine Tamponade mit Calciumalginat oder Auflegen adrenalingetränkter Kompressen hilfreich sein. Der Patient sollte über das postoperativ notwendige Verhalten wie Lagerung, körperliche Schonung, Sportverbot, Zeitpunkt des Fadenzugs, ausreichend informiert werden. In einer retrospektiven Studie an 866 biopsierten Patienten mit Unterschenkelwunden konnten Baraldi et al. [2]. zeigen, dass bis auf Schmerzen während des Eingriffes die oben beschriebenen Komplikationen nur in wenigen Einzelfällen auftraten.

## Fazit für die Praxis

Die Aufarbeitung einer Biopsie stellt eine wichtige Säule in der Diagnostik chronischer Wunden dar und ergänzt die Basisdiagnostik sowie das strukturierte Assessment chronischer Wunden. Je nach möglicher Verdachtsdiagnose kommen dabei unterschiedliche Entnahmetechniken und histologische oder mikrobiologische diagnostische Aufarbeitungen infrage. Dabei ist es erforderlich, je nach Fragestellung, die Probeentnahme korrekt durchzuführen, das Präparat mit einer zielgerichteten Fragestellung an das Labor weiterzuleiten und das Ergebnis der Diagnostik zusammen mit Anamnese und Klinik einzuordnen. Auch bei fehlendem Ansprechen auf eine vermeintlich adäquate Therapiemaßnahme ist eine Biopsie erforderlich, um die bisherige Arbeitsdiagnose zu überprüfen. Um eine frühzeitige Diagnostik und Einleitung kausaler Therapiemaßnahmen zu fördern, ist eine zeitnahe Zuweisung von Patienten mit chronischen Wunden an ein spezialisiertes Zentrum erforderlich. Eine interdisziplinäre Zusammenarbeit der unterschiedlichen Fachrichtungen kann dabei die schnelle Versorgung unterstützen.
